# YOLOv11-GSF: an optimized deep learning model for strawberry ripeness detection in agriculture

**DOI:** 10.3389/fpls.2025.1584669

**Published:** 2025-08-20

**Authors:** Haoran Ma, Qian Zhao, Runqing Zhang, Chunxu Hao, Wenhui Dong, Xiaoying Zhang, Fuzhong Li, Xiaoqin Xue, Gongqing Sun

**Affiliations:** College of Software, Shanxi Agricultural University, Taigu, China

**Keywords:** object detection, strawberry, YOLOv11, ghost module, C3K2-SG module, F-PIoUv2 loss function

## Abstract

The challenge of efficiently detecting ripe and unripe strawberries in complex environments like greenhouses, marked by dense clusters of strawberries, frequent occlusions, overlaps, and fluctuating lighting conditions, presents significant hurdles for existing detection methodologies. These methods often suffer from low efficiency, high computational expenses, and subpar accuracy in scenarios involving small and densely packed targets. To overcome these limitations, this paper introduces YOLOv11-GSF, a real-time strawberry ripeness detection algorithm based on YOLOv11, which incorporates several innovative features: a Ghost Convolution (GhostConv) convolution method for generating rich feature maps through lightweight linear transformations, thereby reducing computational overhead and enhancing resource utilization; a C3K2-SG module that combines self-moving point convolution (SMPConv) and convolutional gated linear units (CGLU) to better capture the local features of strawberry ripeness; and a F-PIoUv2 loss function inspired by Focaler IoU and PIoUv2, utilizing adaptive penalty factors and interval mapping to expedite model convergence and optimize ripeness classification. Experimental results demonstrate the superior performance of YOLOv11-GSF, achieving an average precision of 97.8%, an accuracy of 95.99%, and a recall rate of 93.62%, representing improvements of 1.8%, 1.3 percentage points, and 2.1% over the original YOLOv11, respectively. Furthermore, it exhibits higher recognition accuracy and robustness compared to alternative algorithms, thus offering a practical and efficient solution for deploying strawberry ripeness detection systems.

## 1 Introduction

Strawberry is one of the important high-economic-value fruit in China, with its cultivation area and production volume continuously increasing in recent years (Amr et al., 2020). Pre-harvest yield assessment is a crucial link in strawberry cultivation management, providing vital data support for sales plan formulation and fertilizer application schemes for the next season. Currently, strawberry yield assessment primarily relies on historical yield data and manual statistics, which are inefficient and prone to large errors (Tang et al., 2025). According to the “Series of Reports on China’s Economic and Social Development Achievements in the Past 75 Years,” with the rapid development of information technology, new-generation technologies such as the Internet of Things, big data, and artificial intelligence have gradually been applied to strawberry cultivation and management, driving the intelligent progress of strawberry maturity detection. In the context of smart agriculture, maturity detection technologies based on image recognition and deep learning have gradually gained widespread attention and application. These automated detection technologies not only improve harvesting efficiency and eliminate the inaccuracies of manual judgment, but also provide farmers with precise harvesting time suggestions, reducing strawberry losses. Science and technology for agriculture and assisting agriculture have become the main theme of modern agriculture. The rapid advancement of machine vision and deep learning technologies has led to the increasing maturity of target detection applications in agriculture (Xu et al., 2022). Studies have shown that deep learning technologies can achieve rapid and accurate identification and classification of fruits, providing with more convenient picking and management services (Chen G. et al., 2024). Therefore, for the problems of low efficiency and labor shortage of traditional manual statistics, the automated assessment of strawberry yield through intelligent target detection technology can not only improve the prediction accuracy and timeliness, but also provide technical support for the digital upgrading of the whole process of planting management, which is of great significance in promoting the quality and efficiency of the strawberry industry (Yang et al., 2023).

In recent years, domestic and foreign researchers have conducted extensive research on strawberry recognition. Cui et al. (2025) proposed an improved YOLOv5 target detection algorithm based on the GAM(Global Attention Mechanism) attention mechanism, enhancing the model’s feature extraction capability by adding the GAM attention mechanism to the neck network of the YOLOv5 model, and Analyzing the fusion detection effects of different types of attention mechanisms to optimize the balance between strawberry detection accuracy and efficiency. Gong et al. (2024) designed a strawberry picking robot capable of precise recognition and localization of strawberries, with a recognition rate of 95% for ripe strawberries. Tang et al. (2023) proposed an improved YOLOv7-Tiny model for ripe strawberry recognition. Based on the YOLOv7-Tiny model, this model replaced the Leaky ReLU activation function of the CBL convolutional block in the backbone network with the Sigmoid Linear Unit (SiLU) function, improving the model’s nonlinear fitting degree and feature learning ability. Its recognition results are superior to the original SSD, Faster RCNN, YOLOv3, YOLOv4, and YOLOv5s models. Yang et al. (2024) proposed a strawberry recognition and localization method combining an improved YOLOv8 algorithm with a pose keypoint detection algorithm. This method introduces the Bidirectional Feature Pyramid Network (BiFPN) and GAM modules to enhance the bidirectional information flow of the model, dynamically allocate feature weights, and focus on the extraction of small target features and the enhancement of features in occluded areas, aiming to improve the accuracy of picking point localization and the prediction accuracy of occlusion recognition in complex environments. Compared with the original model, the improved YOLOv8-pose model exhibits improvements of 6.01%, 1.98%, 6.67%, and 7.85% in strawberry recognition accuracy, recall rate, average precision, and keypoint average precision, respectively.

Currently, in terms of the selection of cultivation methods, most models focus on identifying ripe strawberry grown in the ground (Zhao and Cui, 2024). This study takes the growth characteristics of elevated Kanoya Strawberry (Sui Zhu Strawberry) as the research object, emphatically analyzing the two critical stages of strawberry swelling and ripe coloring for automated management and yield prediction. Farmers can promptly adjust the environmental parameters of elevated cultivation (such as light intensity, temperature, humidity, nutrient solution concentration, etc.) by monitoring changes in strawberry diameter and coloring area. This allows them to adopt precise management measures, such as optimizing water and fertilizer coordination and control, supplementing CO_2_ gas fertilizer, or adjusting spectral lighting, thereby simultaneously enhancing fruit quality and the commercial fruit rate. Based on deep learning analysis of image from these two critical stages, this study aims to construct an identification model for the growth status of elevated strawberry. This provides technical support for automated decisions, such as targeted thinning and partitioned harvesting warnings. As a result, it reduces the frequency of manual inspections. Furthermore, it achieves precise on-demand supply of water, fertilizer, and pesticides. Additionally, it lowers pollution risks and saves costs. Ultimately, this approach promotes the green and low-carbon transformation of elevated strawberry production.

In the realm of algorithms, despite the remarkable progress achieved by the original YOLO object detection model and its various YOLO-based improved algorithms in the agricultural field, they still encounter a series of pressing scientific challenges when detecting the maturity of strawberries in high-rise cultivation environments. For instance, there are significant difficulties in accurately identifying small-sized strawberry targets, insufficient effectiveness in extracting maturity-related features under complex background interferences, and the struggle to achieve an ideal balance between model computational efficiency and detection accuracy. This article will operate with the YOLOv11 model, proposing a series of improvements to the model and loss function for the task of ripeness detection of elevated Zhuisu strawberry. In terms of the model, the GhostConv convolution method is first introduced, which generates more feature maps through low-cost linear transformations, effectively reducing computational burden and improving resource utilization. Secondly, the C3K2-SG module is referenced, which integrates the strengths of self-moving point convolution (SMPConv) and convolutional gated linear units (CGLU). It more effectively captures the local features of strawberry ripeness and enhances target feature extraction capabilities, particularly demonstrating notable advantages when dealing with small targets and complex background interference. In terms of the loss function, an F-PIoUv2 loss function is referenced, which draws on the design ideas of Focal IoU and PIoUv2, accelerating model convergence and optimizing strawberry ripeness detection results through adaptive penalty factors and interval mapping methods. These improvement methods collectively enhance the performance and stability of the YOLOv11 model in strawberry ripeness detection tasks.

## 2 Materials and methods

### 2.1 Data collection

**Figure 1:**
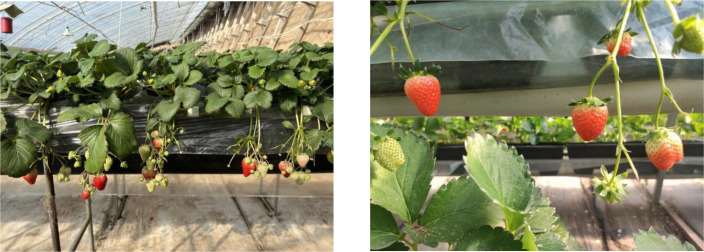
Greenhouse elevated strawberry.

This study adopts elevated planting in solar greenhouses, taking the Kanoya Strawberry as the research object. The solar greenhouse covered an area of 0.13 hectares and is planted with a total of 15,000 strawberry plants. The row spacing is 1.5 meters, and the plant spacing is 15 centimeters. Compared with traditional ground planting, elevated strawberry planting makes more efficient use of space, as shown in **Figure 1**.

**Figure 2:**
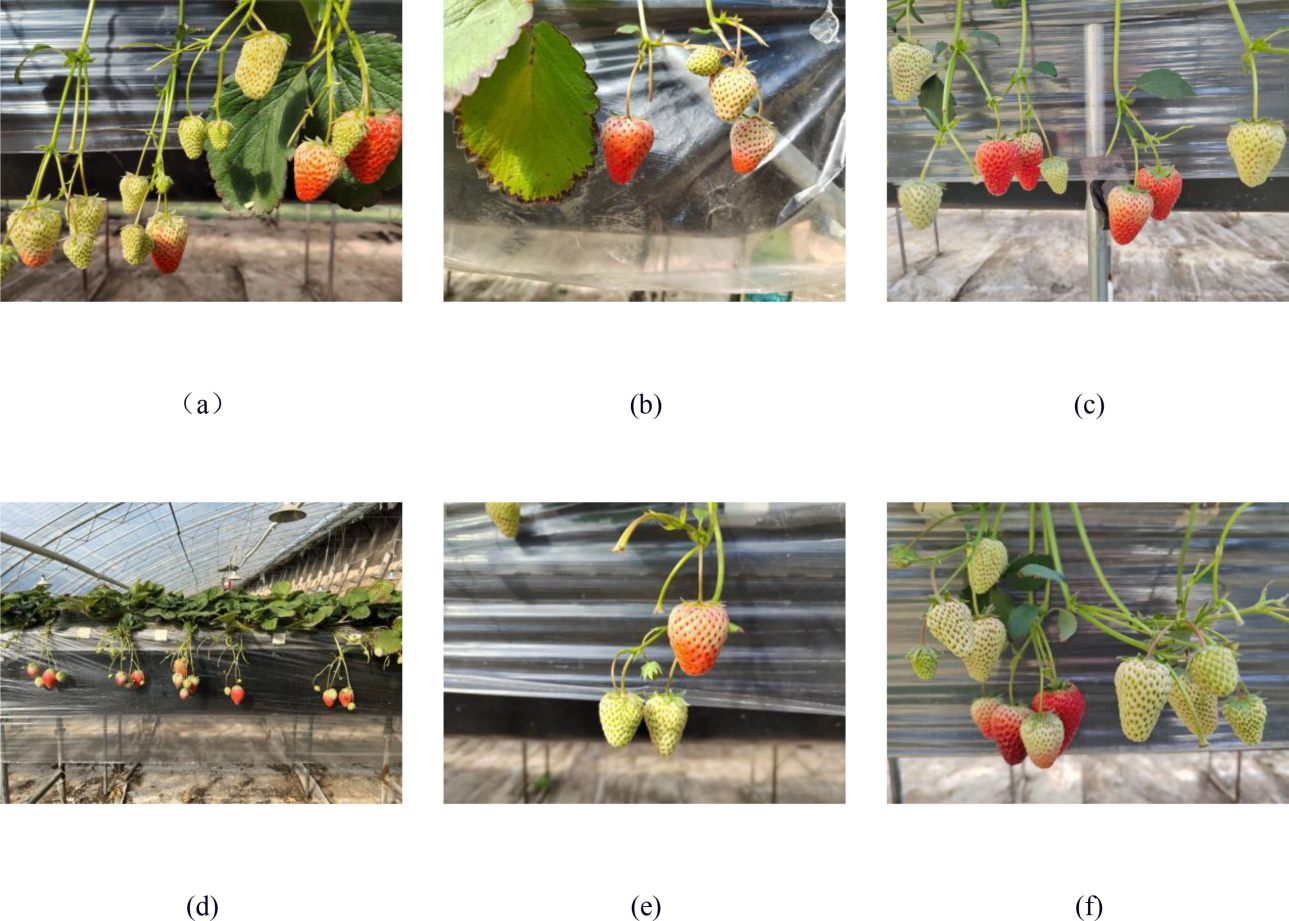
Images of strawberries in different complex scenarios. **(a)** Back light; **(b)** Front light; **(c)** Close-distance; **(d)** Long-distance; **(e)** Unobstructed; **(f)** Obstructed.

Based on improved YOLO series algorithms, strawberry detection is conducted for naturally grown elevated strawberries. Most strawberry are diamond-shaped, varying in size and growing in overlap. Ti Provhe data is collected exclusively from the strawberry experimental field in Xiaowang Village, Xia County, Yuncheng City, Shanxince, ensuring consistency and uniformity in the data source. Data collection is done manually, involving the selection of three time periods during the fruit coloring stage according to different growth cycles of strawberries, and the collection of data from multiple angles, distances, and lighting intensities. Dataset includes backlight, front light, close-up, long-distance, unobstructed, and obstructed views, as shown in **Figure 2**. The collected original images are in.jpg format, with a resolution of 4096*3072 pixels, totaling 2,985 images.

### 2.2 Data processing

In this experiment, LabelImg software is used to perform bounding box labeling on each strawberry in the original images. To focus more on distinguishing whether strawberries are ripe, two data types are established, namely “0” and “1”, corresponding to unripe and ripe, respectively. The data is saved in YOLO training format, generating corresponding TXT files for storing the two-dimensional coordinate information of the objects. Based on actual taste during cultivation, objects in the expansion stage with a coloring area of less than 70% are labeled as “0”, and objects with a coloring area of more than 70% are labeled as “1”.

The dataset is divided into 1,791 training, 896 validation, and 298 test samples (6:3:1 ratio) to balance model training adequacy with generalization assessment, reducing overfitting risk. Each dataset is further divided into two folders: Images and Labels, storing the original.jpg data and corresponding.txt annotation information, respectively. It should be noted that the dataset is currently not publicly available. Interested researchers can contact the authors to request access to the dataset.

**Figure 3:**
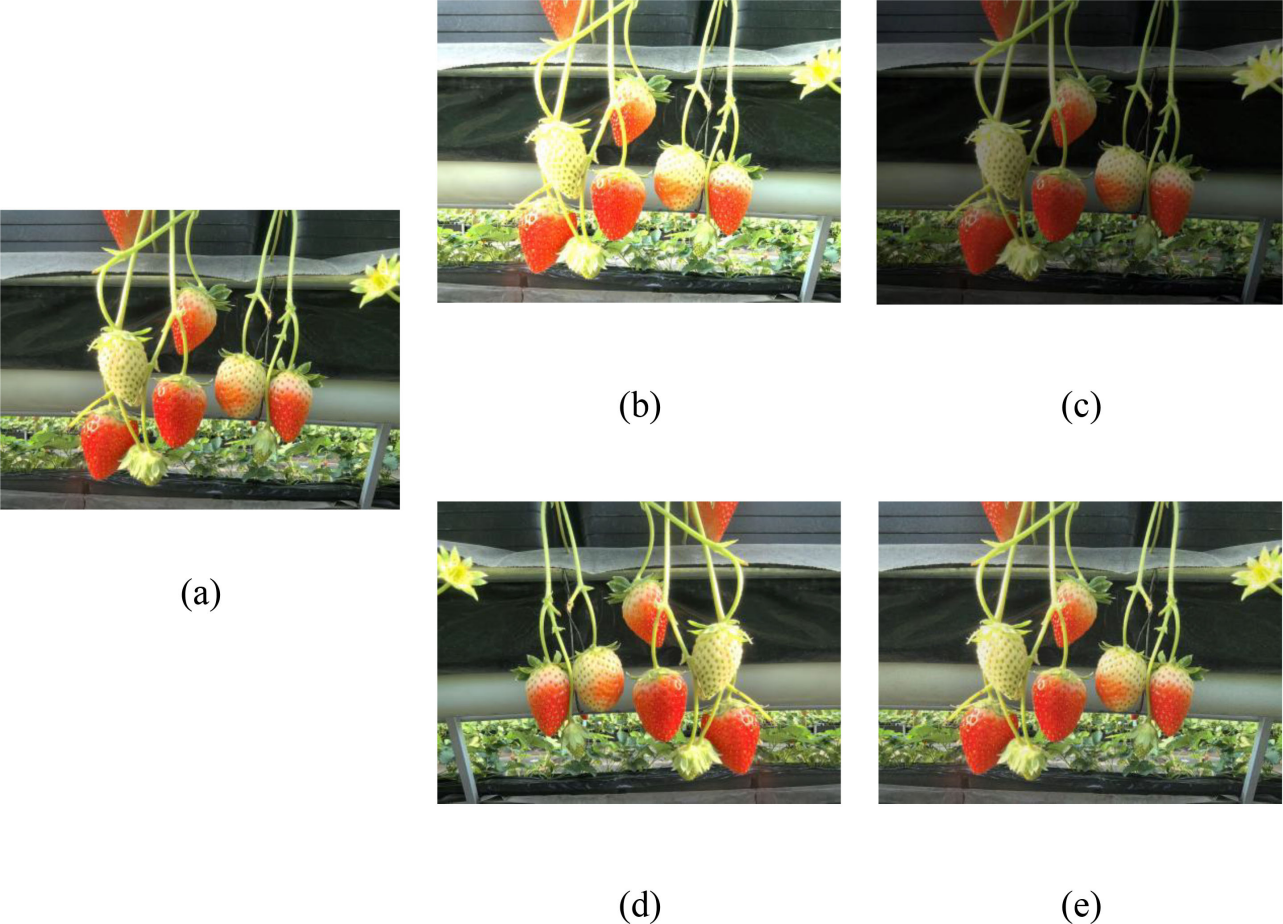
Example of image enhancement. **(a)** original **(b)** random brightness; **(c)** random contrast; **(d)** random flipping; **(e)** random noise.

During the training process, data augmentation is performed using four methods: horizontal flipping, vertical flipping, brightness enhancement, and brightness reduction of the images, as shown in **Figure 3**.

### 2.3 Principle of YOLOv11

**Figure 4:**
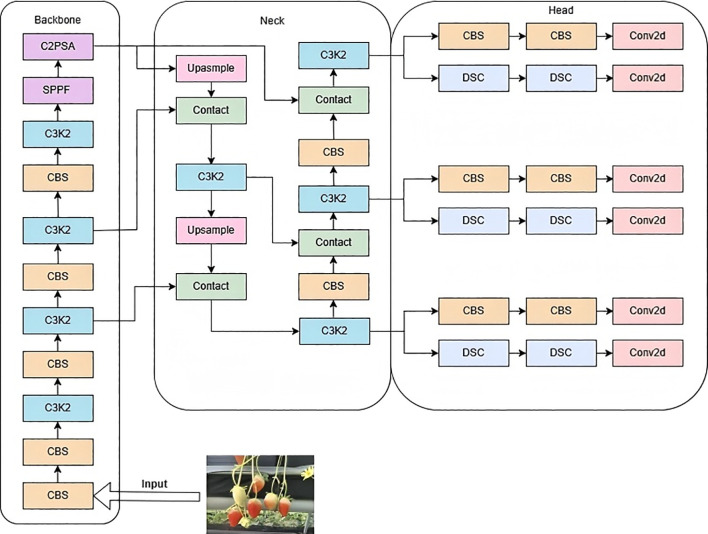
Network architecture of YOLOv11.

YOLOv11 is the latest version of the Ultralytics YOLO series, suitable for various computer vision tasks such as object detection, instance segmentation, image classification, pose estimation, and oriented object detection. The model architecture is divided into three parts: Backbone, Neck, and Head (Ana et al., 2020), as shown in **Figure 4**. Compared with YOLOv8, it uses the C3k2 module to replace the original C2f module, which excels in feature aggregation. The C2PSA module, utilizing a multi-head attention mechanism for global feature extraction, is integrated into the YOLOv11 model, thereby further augmenting its feature extraction capabilities. The detection head adopts advanced technologies such as depthwise separable convolutions (Zhai et al., 2025) and DynamicHead, significantly improving the model’s computational efficiency and detection accuracy. These improvements make YOLOv11’s detection head more flexible and efficient while maintaining high performance. Meanwhile, by optimizing the network architecture and reducing the number of parameters (Zhao and Jia, 2022), YOLOv11 achieves faster processing speeds while maintaining high performance. However, the remarkable progress achieved by YOLOv11 still encounters a series of pressing scientific challenges when detecting the maturity of strawberries in high - rise cultivation environments. Therefore, this study selects YOLOv11 as the baseline model.

### 2.4 Improvements to YOLOv11

**Figure 5:**
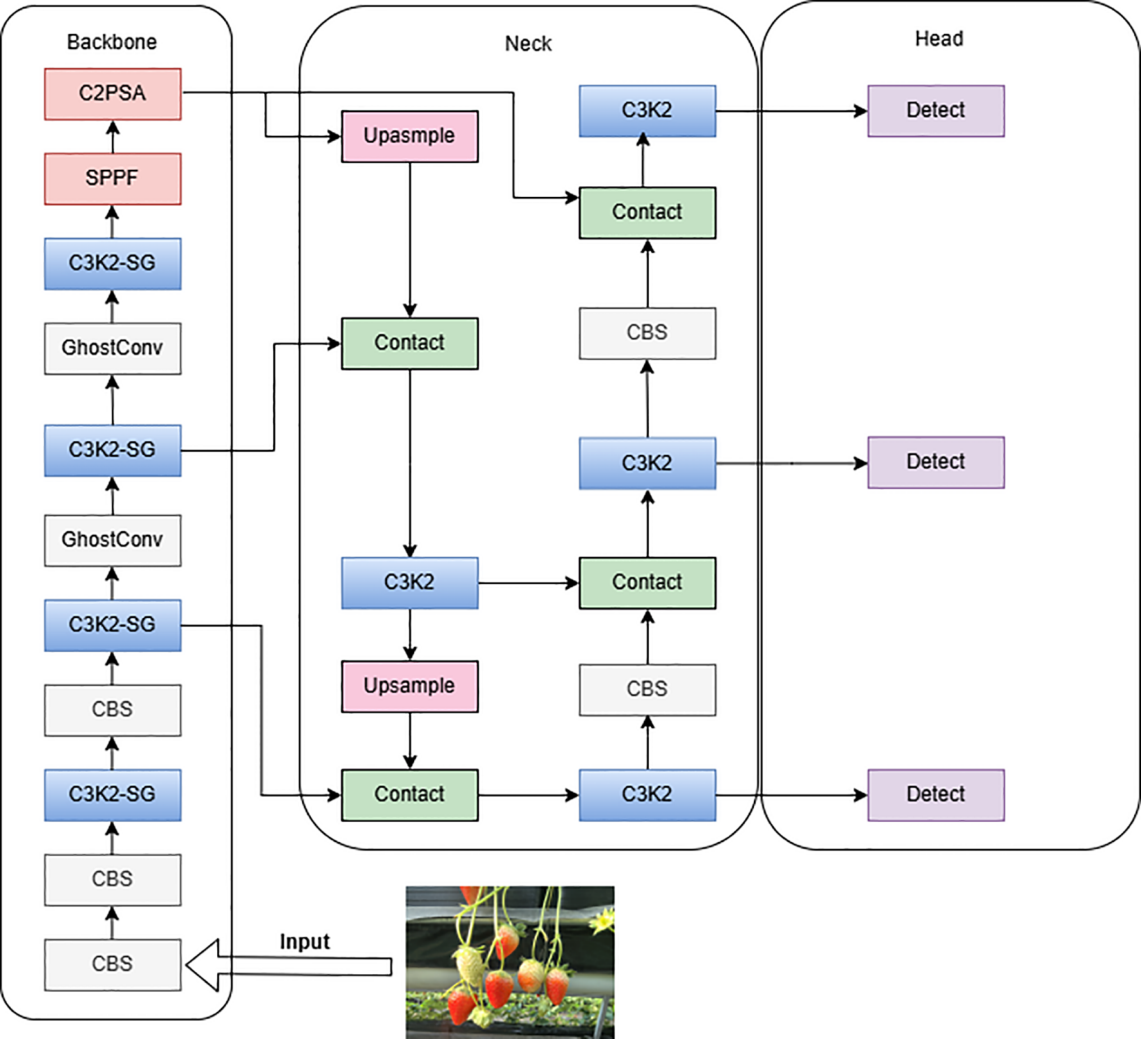
Network architecture of the improved YOLOv11.

Addressing issues such as low detection accuracy, frequent missed detections, and false positives in strawberry ripeness detection for small targets, innovative improvements based on YOLOv11n were proposed to enhance the performance of strawberry maturity detection in greenhouse high-shelf systems. Firstly, GhostConv modules were introduced in the Backbone to replace the fifth and seventh convolutions, reducing computational load by generating “real” and “ghost” feature maps, thereby improving model efficiency. Secondly, the C3K2-SG module was designed within the Backbone, utilizing a point-shifting mechanism to enhance the effectiveness and flexibility of feature extraction, facilitating accurate identification of target defects against complex backgrounds. Lastly, the F-PIoUv2 loss function was developed to improve model detection performance by focusing on different regression samples. The improved YOLOv11 model is illustrated in **Figure 5**.

#### 2.4.1 GhostConv

GhostConv, also known as Ghost convolution or phantom convolution, is an efficient convolution method aimed at generating more feature maps through low-cost linear transformations to reduce computational burden and improve resource utilization (Si et al., 2024). In well-performing CNN models, feature map redundancy is crucial (Lü et al., 2022). Many output features are similar and can be derived through straightforward linear transformations, rather than intricate nonlinear ones. Therefore, one feature map can be considered as the “ghost” of another. A structure that generates a large number of feature maps through a small amount of computation is called a Ghost Module. This structure generates feature maps through a series of linear operations, where the feature maps generated through linear operations are called ghost feature maps, and the feature maps being operated on are called intrinsic feature maps.

(1) Implementation of GhostConv

Regular Convolution: Perform regular convolution operations on the input feature maps to obtain a set of intrinsic feature maps( $Y^{\prime }$ ). The calculation is as Equation 1.


(1) Y'=X*f'

Ghost Generation: Based on the obtained intrinsic feature maps, apply a series of simple linear operations ( $\Phi_{\text{ij}}$ ) to generate ghost feature maps ( $Y_{\text{ij}}$ ). The calculation is as Equation 2. These linear operations are usually low-cost as they do not require additional convolutional kernels and significant computational resources.


(2) $${\text{Y}}_{ij}=\Phi_{i,j}({Y^{\prime }}_{i}),\forall i=1,\ldots ,m,j=1,\ldots ,s$$

Feature Map Concatenation: Concatenate the intrinsic feature maps and the generated ghost feature maps in the channel dimension to obtain the final output feature maps.

(2) Advantages of the Ghost Module

Using fewer convolutions, for example, with 64 convolutional kernels, this structure only needs 32, reducing the computation by half.

**Figure 6:**
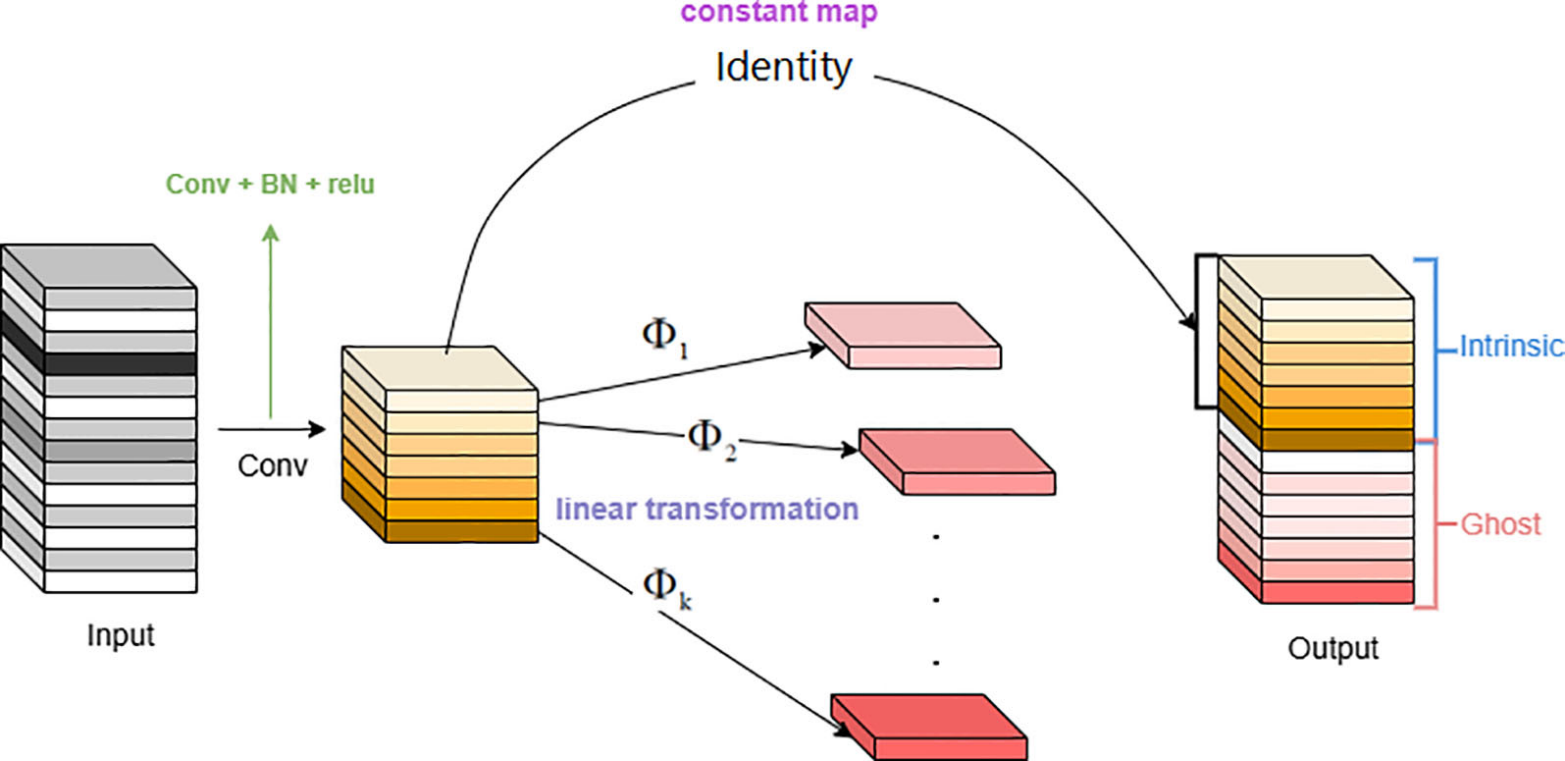
The ghost module.

The low-cost operations ( Φ ) include a series of 3×3 and 5×5 convolutional kernels (Liu et al., 2025), and the convolutions are performed on a per-feature-map basis, as shown in **Figure 6**.

Assuming the shape of the input feature map is [32, 32, 6], a 1x1 convolution is applied to reduce the number of channels, resulting in a shape of [32, 32, 3]. Then, a 33 depthwise convolution is used to extract features from each channel’s feature map, maintaining the shape of [32,32,3], which can be regarded as a series of linear transformations obtained from the previous layer. Finally, the output feature maps from the two convolutions are stacked along the channel dimension, resulting in a shape of [32,32,6].

(3) Conv-Ghost Param Ratio

The ${\text{r}}_{s}$ represents the ratio of the computational complexity of ordinary convolution operations to that of the Ghost module. The calculation is as Equation 3.


(3) $$\begin{array}{c} {\text{r}}_{\text{s}}=\frac{n\cdot h^{\prime }\cdot w^{\prime }\cdot c\cdot k\cdot k}{\frac{n}{s}\cdot h^{\prime }\cdot w^{\prime }\cdot c\cdot k\cdot k+(s-1)\cdot \frac{n}{s}\cdot h^{\prime }\cdot w^{\prime }\cdot d\cdot d}\\ =\frac{c\cdot k\cdot k}{\frac{1}{s}\cdot c\cdot k\cdot k+\frac{s-1}{s}\cdot d\cdot d}\approx \frac{s\cdot c}{s+c-1}\approx s \end{array}$$

The c represents the number of input channels, h is the height of the input image, w is the width of the input image, h′ is the height of the output image, w′ is the width of the output image, k is the size of the convolutional kernel, d is the size of the convolutional kernel for linear transformation, n is the number of output channels, s is the number of transformations, and n\s is the number of output channels after the first transformation.

The ‘s-1’ arises due to the fact that the identity mapping, though involving no computation, is counted as part of the second transformation, consequently lowering the computational complexity of the Ghost module.

(4) Conv-Ghost Complexity Ratio

The ${\text{r}}_{c}$ represents the ratio of the computational complexity of ordinary convolution operations to the number of parameters in the Ghost module. The calculation is as Equation 4.


(4) $${\text{r}}_{\text{c}}=\frac{n\cdot c\cdot k\cdot k}{\frac{n}{s}\cdot c\cdot k\cdot k+(s-1)\cdot \frac{n}{s}\cdot d\cdot d}\approx \frac{s\cdot c}{s+c-1}\approx s$$

The other characters in the formula are consistent with those in Equation 3.

By utilizing existing feature maps to generate more Ghost feature maps, GhostConv significantly reduces the required computational complexity and the number of parameters. This approach also maximizes the utilization of available computing and memory resources, making it particularly suitable for resource-limited embedded systems.

#### 2.4.2 C3k2-SG module

In current agricultural production, detecting strawberry ripeness is challenging due to small target size, variable image quality, and environmental factors. Traditional convolutional neural networks often struggle, as excessive convolution can lead to redundant features and reduced accuracy for small targets like strawberries.

To address this, the study introduces the C3K2-SG convolutional neural network module, designed to enhance local feature extraction for strawberry ripeness detection. The module incorporates a dynamic weight mechanism, allowing selective adjustment and transfer of weight parameters based on information channels. This enables the network to adapt more flexibly to local features, focusing on exploring target features rather than relying solely on global information aggregation. Consequently, the C3K2-SG module significantly improves the extraction of local features related to strawberry ripeness, enhancing model accuracy and robustness.

**Figure 7:**
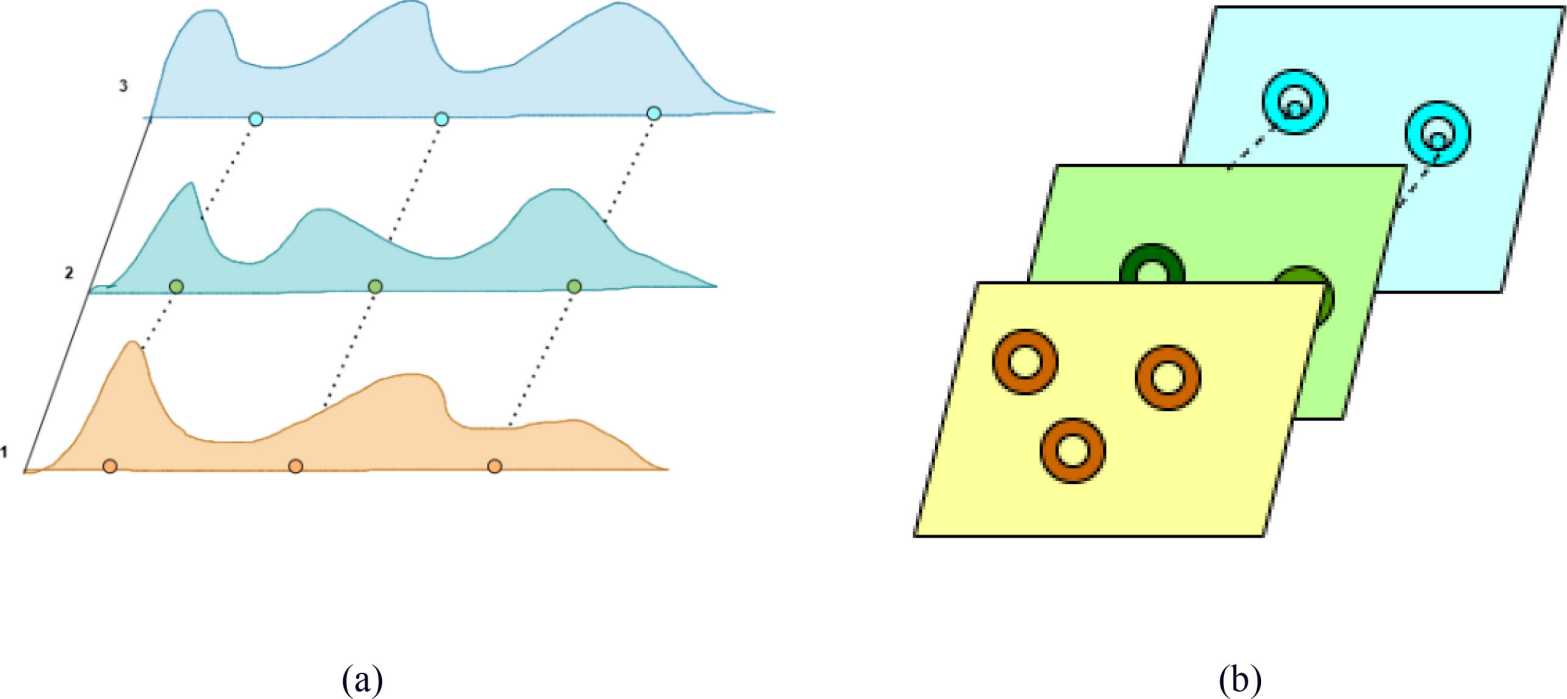
Schematic of SMPConv self-moving points. **(a)** SMP (1D); **(b)** SMP (2D).

The C3K2-SG module combines the C3K2 module with the SG module, which integrates the advantages of Self-moving Points Convolution (SMPConv) (Kim and Park, 2023) and Convolutional Gated Linear Unit (CGLU) (Shi, 2024), to significantly enhance the feature extraction capability of the model for strawberry ripeness detection. Traditional continuous convolution methods typically rely on large convolution kernels, but this approach not only incurs high computational costs but also involves complex hyperparameter tuning and is inefficient. To overcome these issues, SMPConv adopts an innovative approach, as shown in **Figure 7**. SMPConv allows each channel to share points at the same position, while each channel still retains its own independent weight parameters. This mechanism enables SMPConv to effectively control the free movement of weights and achieve continuous convolution through interpolation, thus avoiding the computationally intensive convolution operations found in traditional neural networks. It enhances the flexibility and efficiency of feature extraction, maintaining strong feature extraction capabilities even in complex and diverse data environments.

On the other hand, in the gating branch of GLU, a simplified channel mixing structure is formed by adding a 3×3 depthwise convolution operation before the activation function. This design aligns with the concept of gated channel attention, enabling the model to effectively capture and integrate important information from neighboring image features, thus improving the model’s robustness in various scenarios. Through this channel mixing strategy, the model is able to maintain high accuracy and adaptability when processing complex data.

**Figure 8:**
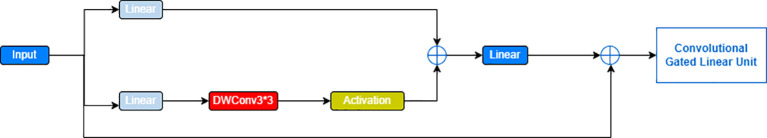
CGLU modules structure diagram.

**Figure 9:**
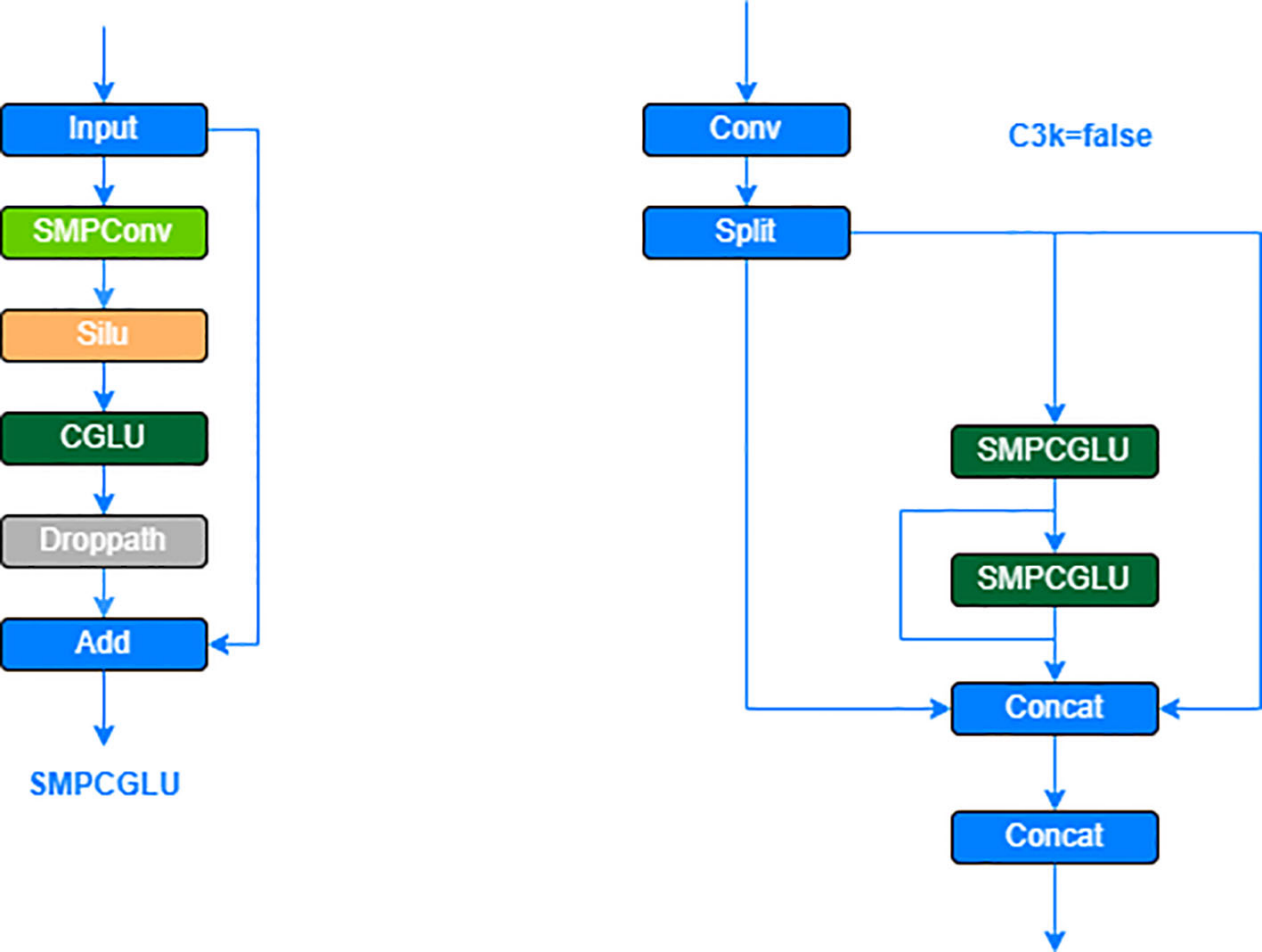
SMPCGLU and C3K2-SG module architecture.

The structure of the SG module is achieved by stacking the CGLU module after the SMPConv. This design not only effectively captures the features of strawberry ripeness but also integrates a gating mechanism to enable selective information flow through the channels, thereby enhancing the flexibility and effectiveness of feature extraction. The specific structure of the CGLU modules is shown in **Figure 8**. module, when the C3K2 parameter is set to false, the SG module replaces the original Bottleneck module. This approach ensures that the model remains lightweight while maintaining effective information flow, ultimately improving the overall performance of the strawberry ripeness detection model (Zhai et al., 2025). The specific structure of the C3K2-SG module is shown in **Figure 9**.

#### 2.4.3 F-PIoUv2 loss function

In the task of strawberry ripeness detection, the collected images often suffer from a certain degree of blurriness, particularly in areas where the color differences in the strawberries are significant. Additionally, the presence of complex backgrounds further complicates the detection process. In traditional loss functions, some penalty mechanisms can cause unnecessary expansion of anchor boxes during the regression process, which slows down the model’s convergence. To address this issue, this study innovatively introduces the F-PIoUv2 loss function, which integrates the design concepts of Focaler IoU (Zhang and Zhang, 2024) and PIoUv2 (Liu et al., 2024). Specifically, PIoUv2 effectively resolves the problem of anchor boxes excessively expanding during regression towards the target location, accelerating the model’s convergence speed. Meanwhile, Focaler IoU alleviates the negative impact of sample imbalance and the varying difficulty of samples on bounding box regression. The F-PIoUv2 loss function, employing an adaptive penalty factor and interval mapping strategy, not only speeds up the model’s convergence but also improves the strawberry ripeness detection performance. The Focaler IoU function, as shown in Equation 5, is constructed using a piecewise function to improve the regression of bounding boxes. The Focal IoU function is shown in Equation 5, which adopts a piecewise function form to construct IoU, thereby improving bounding box regression performance.


(5) $$L_{I\text{o}Ufocaler}=\{\begin{array}{cc} \begin{array}{c} 0,\\ \frac{L_{IoU}-d}{u-d},\\ 1, \end{array} & \begin{array}{c} L_{IoU}<\text{d}\\ d\leq L_{IoU}\leq u\\ L_{IoU}>u \end{array} \end{array}$$

In Equation 5, L_IoU_ refers to the original IoU value, while both d and u belong to the interval from 0 to 1. Different regression samples correspond to different d and u values. The loss(*X_IoUfocaler_*) is defined as shown in Equation 6.


(6) $$X_{I\text{o}Ufocaler}=1-L_{IoUfocaler}$$

To put it simply, by observing the Focaler IoU formula, we can see that when $L_{I\text{o}U}$ is below a certain specific value, the value of $L_{I\text{o}U}$ focaler becomes zero. By examining the Focaler IoU formula, it can be observed that the value becomes zero when it is less than a certain threshold. In the strawberry ripeness detection, where small object detection is required in complex environments, the original design does not fully meet the needs. Therefore, this study improves the Focaler IoU by removing the original two parameters( d and u ), while still maintaining the piecewise function form. This modification enhances the model’s ability to fully utilize sample data. Consequently, the improved approach enables the model to perform better when detecting small strawberries in complex environments, while also enhancing the model’s stability and convergence (Mao et al., 2023). The definition of the improved loss function is detailed in Equations 7 and 8.


(7) $$L_{I\text{o}U^{f}}=\{\begin{array}{c} \frac{L_{I\text{o}U}}{u},L_{IoU}\leq \text{u}\\ 1,\;L_{IoU}>\text{u} \end{array}\}$$

(8) $$X_{IoU}=1-L_{I{\text{oU}}^{f}}$$

Many traditional loss functions guide anchor boxes to move closer to the ground truth boxes by first gradually expanding the anchor boxes until they completely cover the true boxes, followed by a shrinking operation to achieve linear regression (Sun et al., 2024). In contrast, the PIoU loss function effectively addresses the problem of anchor box expansion. It could adaptively select penalty factors based on the target size and can flexibly adjust gradients based on the quality of the anchor boxes. This enables the anchor box to directly minimize the distance between its four sides and the ground truth box, efficiently moving along an almost linear trajectory to the position of the ground truth box. Therefore, compared to other IoU loss functions, the PIoU loss function can perform linear regression more quickly. The PIoU loss function as shown in Equations 9 and 10.


(9) $$\text{p}=(\frac{dw_{1}}{w_{gt}}+\frac{dw_{2}}{w_{gt}}+\frac{dh_{1}}{h_{gt}}+\frac{dh_{2}}{h_{gt}})/4$$

(10) $$X_{PI\text{o}U}=L_{IoU}+1-{\text{e}}^{-p^{2}}$$

In the equation: P represents the penalty factor, with the variables defined.

Based on PIoU, a non-monotonic attention layer m(x) is introduced to investigate the focusing mechanism. By combining the attention layer with PIoU, the PIoUv2 loss function is generated. The definitions of the loss functions are shown in Equations 11–13.


(11) $$\text{q}=e^{-p},q\in (0,1]$$

(12) $$\text{m}(x)=3x\cdot e^{-x^{2}}$$

(13) $$X_{PI\text{o}U\text{v}2}=3m(\lambda q)\cdot X_{PI\text{o}U}$$

According to Equation 11, as p increases, q decreases, where q represents the quality of an anchor box. When p equals 0, q is 1, indicating that the ground truth box and the predicted box are perfectly aligned at this point. The hyperparameter λ is used to control the intensity of the attention (Chen Y. et al., 2024).

Combining the Focaler IoU and PIoUv2 loss functions, the F-PIoUv2 loss function is proposed, with its loss definition shown in Equation 14.


(14) $$X_{F-PI\text{o}U\text{v}2}=3m(\lambda q)\cdot (X_{I\text{o}U^{\text{f}}}+1-e^{-p^{2}})$$

The F-PIoUv2 loss function proposed in this study combines the advantages of two loss functions. It not only adaptively selects the appropriate penalty factor based on the target size but also fully considers the overall positional information of the anchor boxes, enhancing the model’s ability to discriminate between anchor boxes of different quality.

## 3 Experimental design

### 3.1 Experimental environment

To ensure fair experimental comparisons, all experiments were conducted on a single computer with a standardized hardware configuration. The system ran Windows 11 Pro and was equipped with an AMD Ryzen 9 7945HX processor with Radeon Graphics, along with an NVIDIA GeForce RTX 4080 GPU. Python 3.10.9 was used as the programming language, and CUDA 11.8 was integrated to accelerate the model training process. For the experimental setup, all input images were uniformly resized to a resolution of 1450×1450 pixels, and each training batch contained 8 images. The model is trained for 200 epochs with a batch size of 16. The initial learning rate is set to 0.01. The optimizer starts with AdamW and later switches to SGD.

### 3.2 Evaluation metrics

To comprehensively assess the performance of the proposed model, this study adopts a series of evaluation metrics, including precision (P), recall (R), average precision (AP), mean average precision (mAP), model size, and detection frames per second (FPS) (Yang et al., 2023). These metrics collectively establish a comprehensive evaluation system aimed at quantifying the model’s efficiency and resource consumption in practical applications. Specifically, precision (P) reflects the accuracy of the model in identifying positive objects, i.e., the proportion of correctly detected targets among all detected targets. Recall (R) reveals the model’s coverage capability for positive samples, expressed as the proportion of correctly detected targets among all actual positive targets. Average precision (AP) provides an effective evaluation means for single-category performance by calculating the average of precision at different recall levels. Mean average precision (mAP), commonly used in multi-category target detection tasks, achieves a comprehensive measure of the model’s overall performance by averaging the AP values of all categories. This study uses mAP50 (IoU threshold of 50%) and mAP50-95 (average mAP across IoU thresholds from 50% to 95% in 5% increments) as evaluation metrics.

In addition, the model size, quantified in megabytes (M), measures the model’s size and memory resource consumption, serving as an important indicator of model complexity. Detection frames per second (FPS) (Di and Qu, 2020), i.e., the number of image frames the model can process per second, directly reflects the model’s detection speed, measured in frames per second. The specific formulas for calculating each evaluation metric are omitted here for brevity (Equations 15–18):


(15) $$P=\frac{TP}{TP+FP}\times 100\%$$

(16) $$R=\frac{TP}{TP+FN}\times 100\%$$

(17) $$AP=\int_{0}^{\;1}P(R)\text{d}R\times 100\%$$

(18) $$\text{F}1=\frac{2*P*R}{P+R}\times 100\%$$

The terms are as follows: TP is True Positive, FP is False Positive, TN is True Negative, and FN is False Negative.

**Figure 10:**
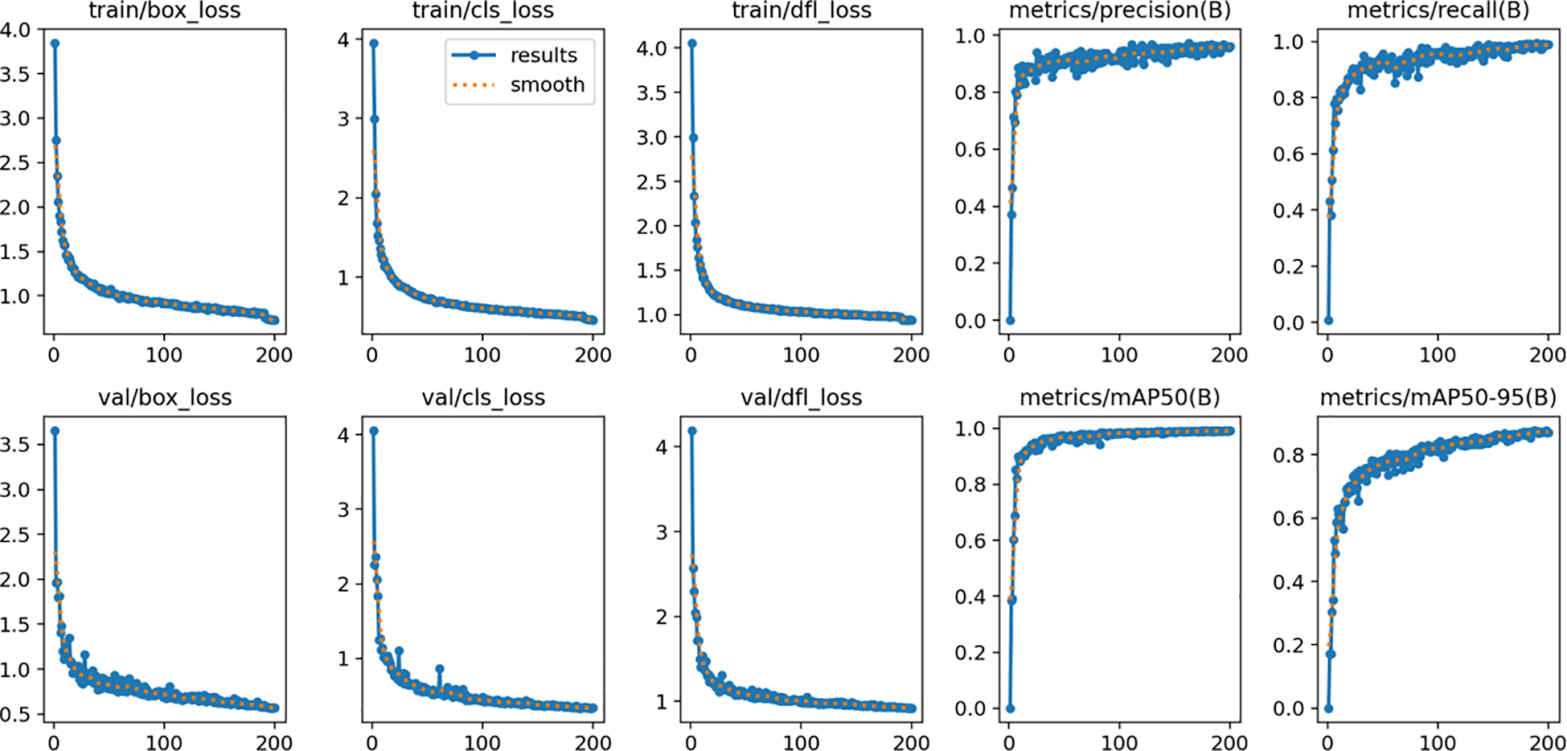
Training loss and evaluation metrics change chart for YOLOv11-GSF.

**Figure 11:**
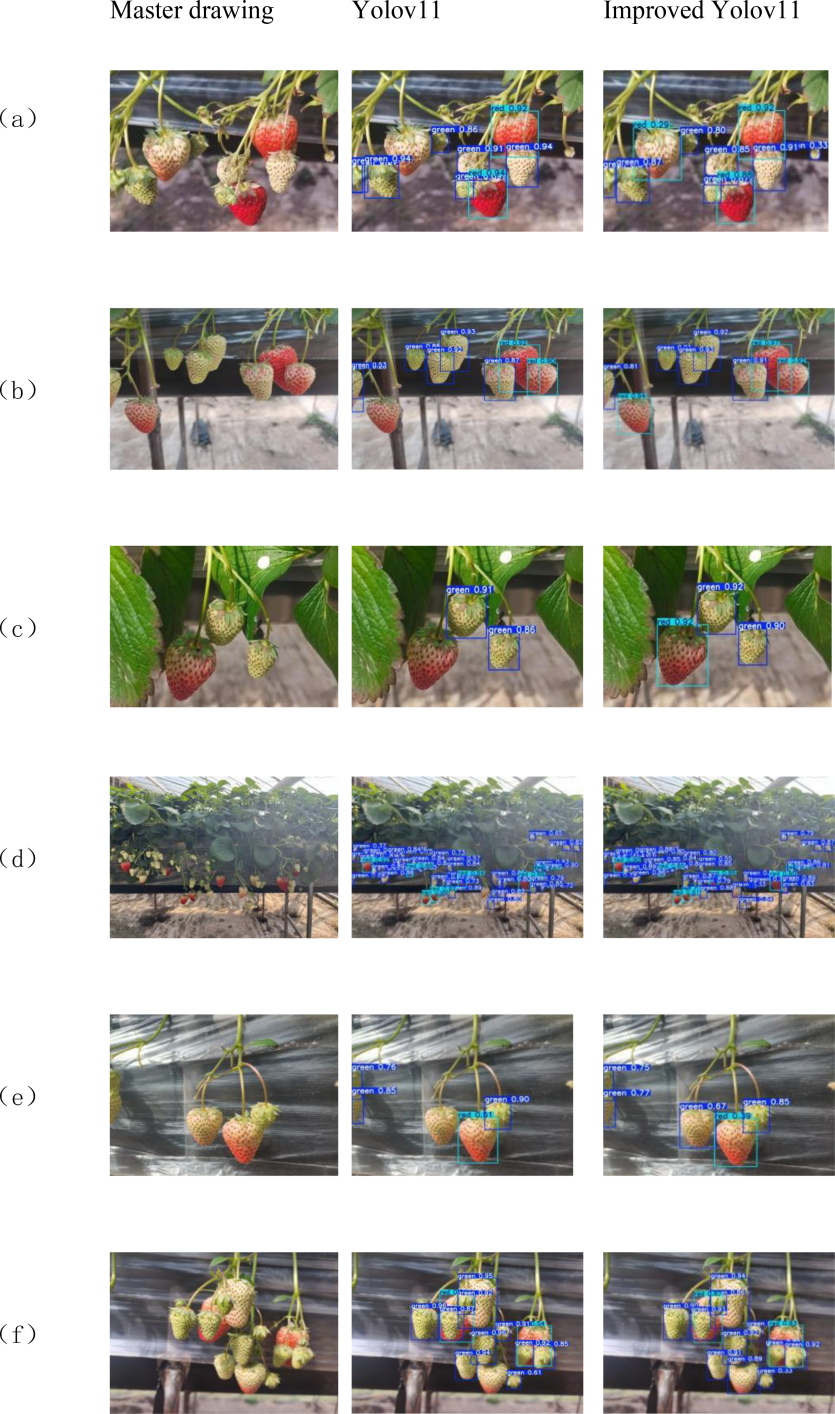
**(a)** Multiple classes and close distance; **(b)** Multiple classes and long distance; **(c)** Simple scenario; **(d)** Small targets; **(e)** Mulching with few fruits; **(f)** Severe occlusion.

To validate the performance of the YOLOv11-GSF model, we meticulously designed a rigorous experimental procedure. The model was trained on a training dataset comprising 1,791 images, fine-tuned on a validation dataset of 896 images, and ultimately evaluated for accuracy using a test dataset consisting of 298 images. The experimental results demonstrated the model’s exceptional performance in strawberry recognition and detection, achieving a precision rate as high as 95.99%, a recall rate of 93.62%, while maintaining a computational efficiency of 6 GFLOPs (Giga Floating-point Operations Per Second) and a FPS of 67.5, which fully underscores the reliability and practicality of YOLOv11-GSF.The false positive rate for unripe fruits is 29.03%, and the false negative rate is 2.94%, while for ripe fruits, the false positive rate is 4%, and the false negative rate is 3.79%. The model performs well in identifying ripe strawberries but exhibits a higher rate of misclassification for unripe ones. **Figure 10** vividly illustrates the improvement effects of YOLOv11 in strawberry detection, presenting a visual comparison that highlights the algorithm’s enhancement in localization accuracy and recognition efficacy. Meanwhile, **Figure 11** quantifies the test accuracy of different experimental models, providing an objective basis for assessing algorithm performance and further substantiating the superiority of YOLOv11-GSF.

## 4 Discussion

### 4.1 Comparison

**Figure 12:**
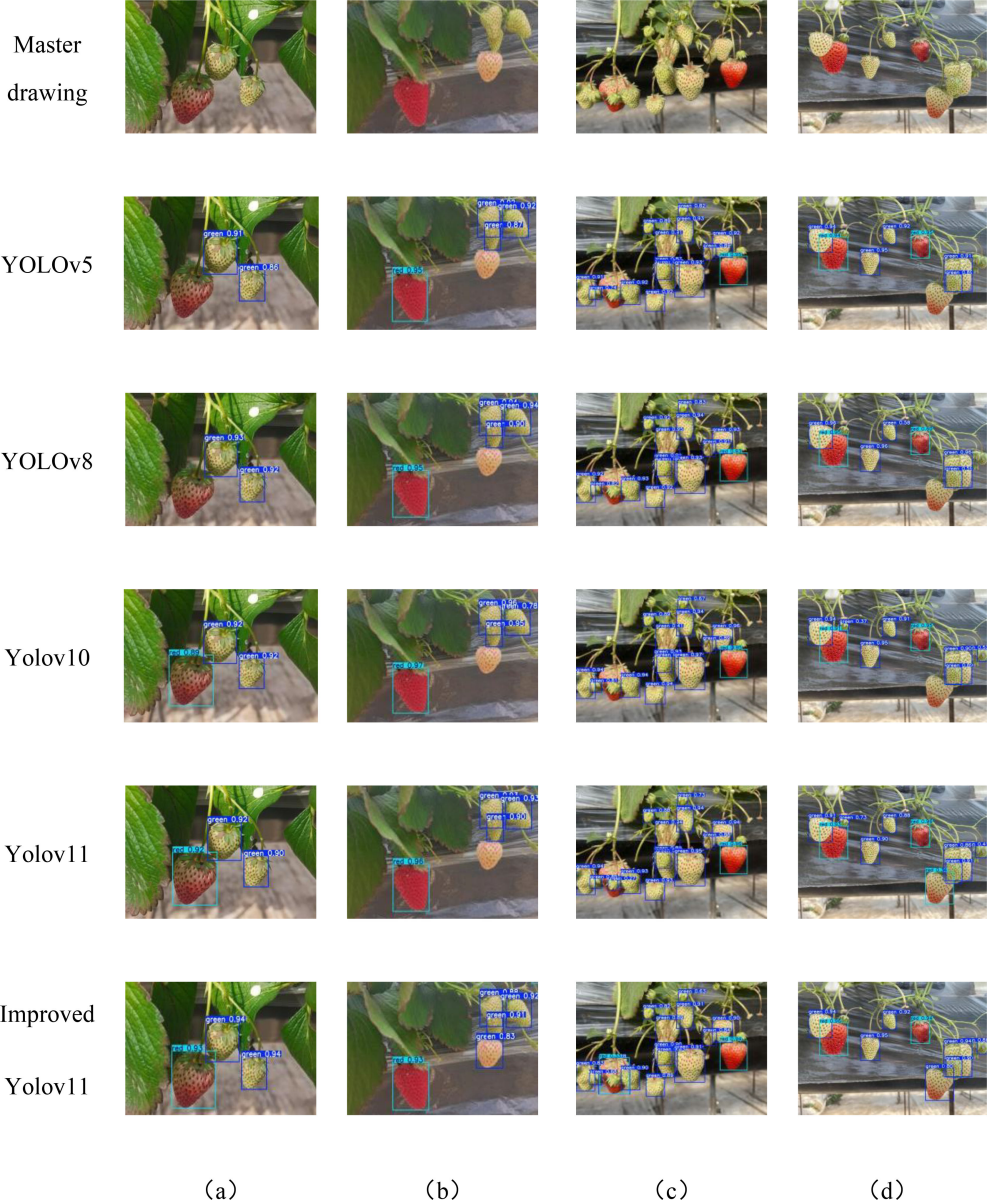
Detection results of different models with various conditions **(a)** Front light and unobstructed ; **(b)** Back light; **(d)** Close-distance and Obstructed; **(c)** Long-distance.

In order to further verify the superiority and effectiveness of the proposed YOLOv11-GSF model in the task of strawberry ripeness target detection, we carried out a series of comparative experiments. These experiments use industry-representative target detection models, i.e., YOLOv5, YOLOv8, YOLOv10, and YOLOv11. The experimental results are illustrated in **Figure 12**. Through comparing the performance of the YOLOv5, YOLOv8, YOLOv10, and the original YOLOv11 models with that of the improved YOLOv11 model in terms of key metrics, including recognition accuracy, recall rate, and average precision, the advantages and effectiveness of each model in the task of ripe strawberry target detection are demonstrated. Moreover, these metrics also highlight the performance disparities among the algorithms when dealing with the ripe strawberry target detection task.

Comparative evaluation of the post-detection output images revealed that the four detection models each exhibited measurable detection performance. Among them, YOLOv11 is outstanding in improving the accuracy of the bounding box, and its bounding box completeness and accuracy outperforms the other models, all of which have varying degrees of deficiencies in detecting the completeness of the annotations. In this case, YOLO5, YOLOv8 and YOLOv10 failed to detect all targets completely, whereas YOLOv11 successfully detected all targets but with recognition misclassification. In the long-range small target detection effect image, the feature extraction capability is significantly enhanced by designing the C3K2-SG module, as shown in **Figure 12D**. This shows that the improved YOLOv11 can not only effectively prevent small targets from being missed in long-distance images, but also improve the detection accuracy, in addition, the improved algorithm shows the best detection performance when dealing with dense images. In conclusion, the improved YOLOv11 demonstrates excellent accurate recognition capabilities in various environments such as long-distance, near-distance and dense scenes. In conclusion, the improved YOLOv11 demonstrates excellent accurate recognition in a variety of environments such as far, near and dense scenes.

**Figure 13:**
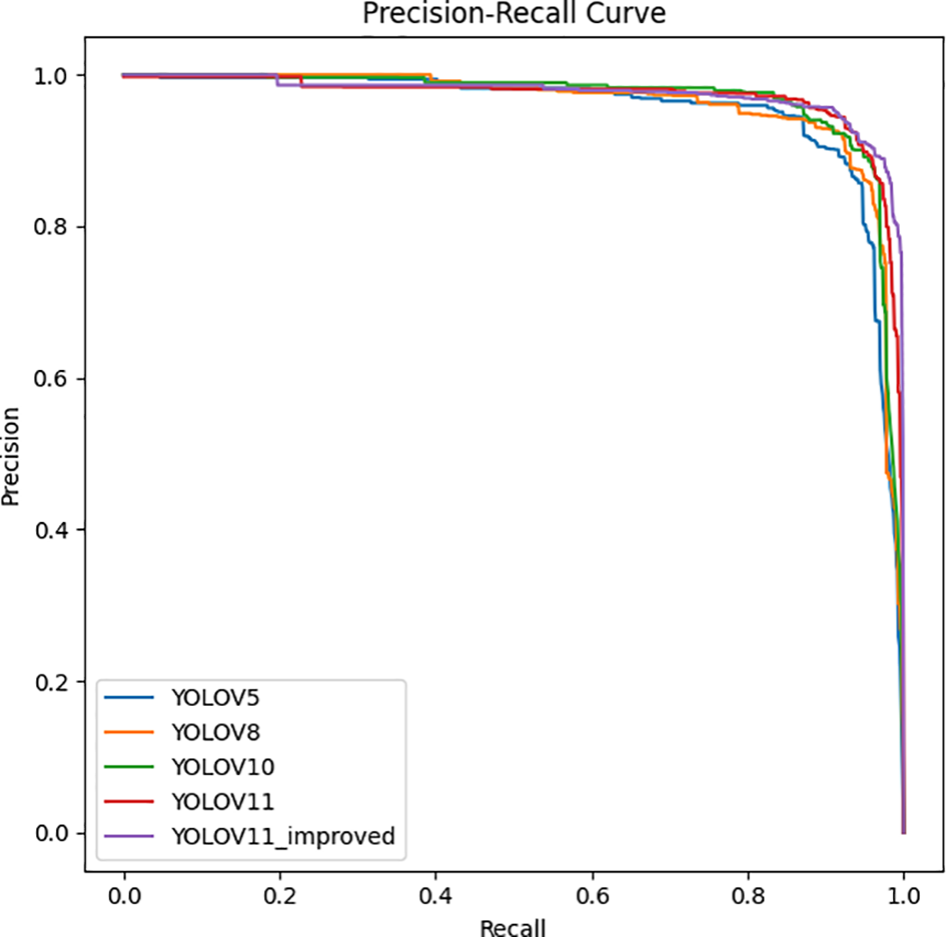
PR curves of different detection models.

As shown in **Table 1**, The experimental results demonstrate that YOLOv11-GSF outperforms the YOLOv11 model in terms of Average Precision, Precision, Recall, and F1 Score. Specifically, YOLOv11-GSF achieves substantial improvements in strawberry ripeness detection, with an Average Precision of 97.8%, a Precision of 95.99%, a Recall of 93.62%, and an F1 Score of 94.79%. Additionally, the model achieves a mean Average Precision (mAP50) of 0.973 at an IoU threshold of 0.5 and a mean Average Precision (mAP50-95) of 0.824 across IoU thresholds from 0.5 to 0.95, further demonstrating its exceptional detection capabilities and robustness. By referring to **Figure 13**, we can observe more intuitively that YOLOv11-GSF, compared to the original network, not only achieves more precise localization of strawberries but also exhibits stronger classification capabilities. This implies that the model can more reliably identify and distinguish strawberries at different stages of maturity. These improvements lay a solid technological foundation for automated strawberry harvesting technology, with the potential to enhance harvesting efficiency and reduce human errors.

**Table 1:** Performance comparison table of YOLO model versions.

Model	Average precision	Precision	Recall(%)	F1 Sore	mAP50	mAP50-95	GFLOPs
Improved YOLOv11	97.8	95.99	93.62	94.79	0.973	0.824	6
YOLOv11	96.8	92.5	92.8	92.64	0.968	0.8	6.6
YOLOv10	96.6	90.9	93	91.93	0.966	0.802	24.8
YOLOv8	96	90.3	93.4	91.82	0.96	0.777	8.9
YOLOv5	95.4	89.9	90.9	90.39	0.954	0.797	4.5

### 4.2 Ablation study

As shown in **Table 2**, the ablation study unveiled the distinct influences exerted by various enhanced modules on the performance metrics of the YOLOv11 model.

**Table 2:** Ablation study performance comparison table for models.

Model	Average precision	Precision	Recall(%)	F1 Sore	GFLOPs
YOLOv11	96.8	92.5	92.8	92.64	6.6
YOLOv11_GhostConv	97.1	93.1	93.6	93.35	6.1
YOLOv11_SG	95.9	93.2	92.5	92.85	6.3
YOLOv11_F-PIoUv2	96.9	92.7	92.9	92.80	6.4
Improved YOLOv11	97.8	95.99	93.62	94.79	6

The GhostConv module demonstrates effective reduction in computational complexity, as evidenced by the decreased GFLOPs, through the generation of simplified feature maps. This module enhances operational efficiency while preserving detection accuracy, rendering it particularly advantageous for mobile platforms with computational constraints. The C3K2-SG module, employing a spatial group convolution strategy, substantially augments feature extraction capabilities. Despite a marginal fluctuation in average precision, this module exhibits distinctive superiority in detecting densely packed small targets. The F-PIoUv2 loss function, by optimizing the bounding box regression mechanism, elevates target localization precision, enabling the model to delineate targets with greater accuracy in complex environments.

Integrated application of all three modules yields synergistic benefits: enhanced detection accuracy and localization efficiency under computational constraints, particularly in challenging environments with distant small targets and dense scenes. This collaborative optimization overcomes individual limitations, providing robust solutions for complex detection tasks.

### 4.3 Visualization

**Figure 14:**
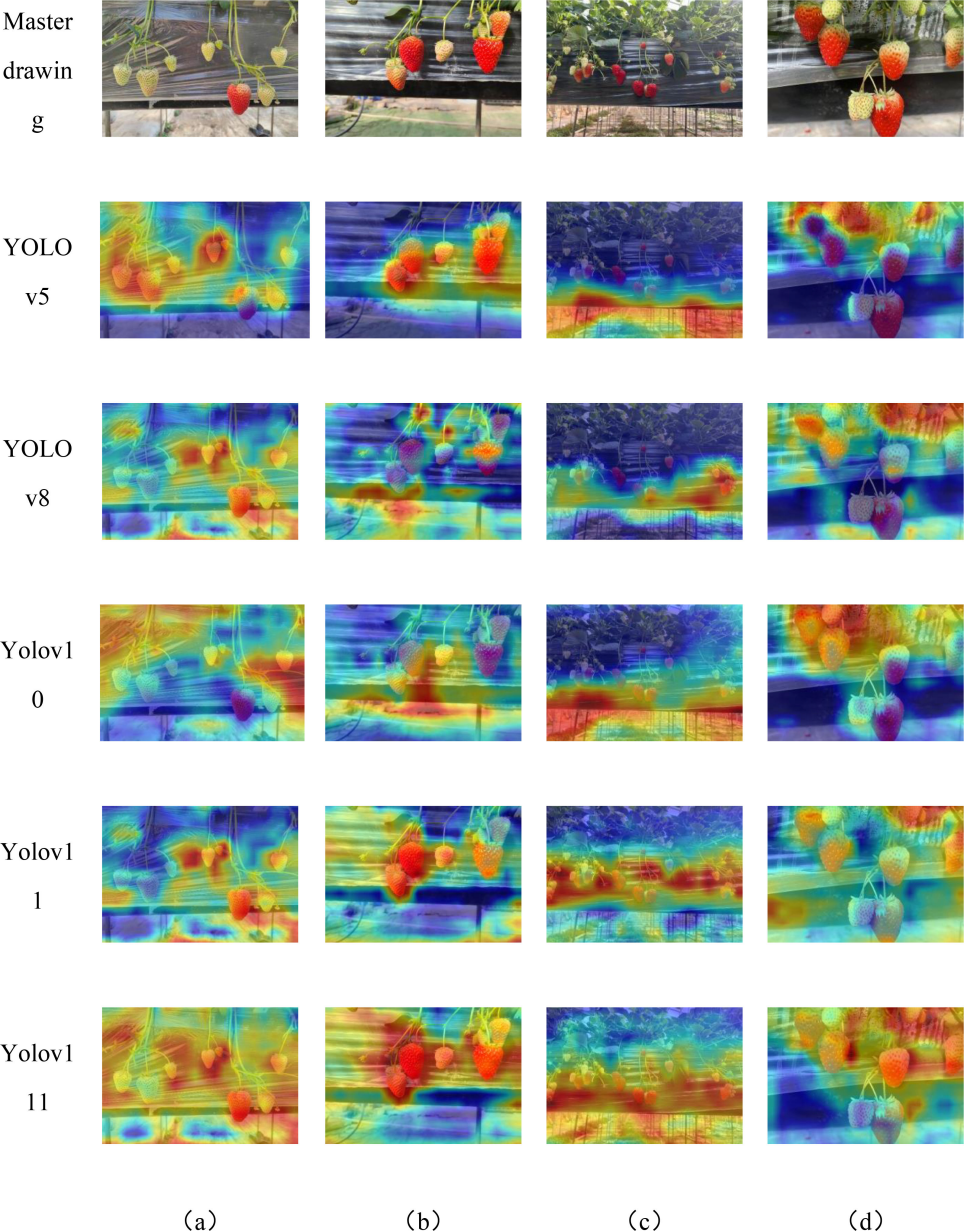
Heatmaps results of different models with various conditions .**(a–d)** are heatmap of different strawberry morphologies.

In order to verify the effectiveness of the proposed model method in strawberry ripeness detection, the optimized network and the original network were compared in a heat map visualization, as shown in **Figure 14**, where the highlighted areas represent the key parts that the network focuses on, and it can be seen that the improved model is able to increase the model’s focus on the strawberry.

The features of strawberry data were visualized by comparing the YOLOv5, YOLOv8, YOLOv10, and YOLOv11 models with the improved YOLOv11 model, using GradCAM visual heat maps. GradCAM visualized and analyzed the output layer, where the red areas indicate the regions that the model focuses on for strawberry detection. The heat maps of all these models are presented in **Figure 14**, enabling a clear visual comparison. The improved YOLOv11’s attention to both ripe and unripe strawberries is improved compared to other YOLO models, which indicates that the improved model can alleviate the influence of the background and better extract the features of the strawberries themselves, proving that the improved model can meet the needs of strawberry ripeness detection. Comparing with the Grad-CAM plots in **Figure 14**, the model with C3K2-SG module extracts more strawberry features compared with the model without C3K2-SG module, which is reflected in the fact that the heat region covers more parts of strawberry region and is brighter and more concentrated.

## 5 Conclusions

This study presents an advanced YOLOv11-based detection framework tailored for strawberry ripeness assessment, demonstrating precise localization of subtle defects in complex fruit imagery. The integration of GhostConv modules into the backbone network—replacing the fifth and seventh convolutional layers—reduces computational complexity through dual-path feature generation. The C3K2-SG module combines self-moving point convolution with adaptive gating mechanisms, enhancing feature extraction flexibility and precision. Additionally, the F-PIoUv2 loss function mitigates penalty factor amplification and class imbalance issues, collectively improving detection performance. Compared to the baseline YOLOv11, the proposed YOLOv11-GSF model achieves a 1% increase in Average Precision (AP), 3.49% higher Precision, 0.82% gain in Recall, and 2.15% elevation in F1 Score, balancing accuracy and real-time efficiency in dynamic agricultural settings.

Despite its algorithmic advancements, the model’s validation remains confined to image datasets, necessitating further real-world testing to ensure operational reliability. Specifically, the framework’s robustness against environmental variability—such as fluctuating lighting conditions, fruit occlusion, and field clutter—requires validation through physical deployment. Planned next steps include comprehensive field trials on unmanned ground vehicles to evaluate performance under natural conditions, coupled with synchronized robotic arm harvesting experiments to assess end-to-end system integration.

Future research will expand this work along three dimensions: (1) edge-device optimization for resource-constrained agricultural robots, (2) seamless integration with automated harvesting systems to enable closed-loop operations, and (3) cross-species adaptability studies to extend the model’s applicability to other small berry crops via transfer learning. By establishing a cohesive “lab-to-field” translational pipeline, this study lays groundwork for scalable, intelligent solutions in precision agriculture.

## Data Availability

The original contributions presented in the study are included in the article/supplementary material. Further inquiries can be directed to the corresponding author.
